# Antimicrobial resistance patterns of *Staphylococcus aureus* isolated from apparently healthy pet cats of Bangladesh

**DOI:** 10.5455/javar.2023.j708

**Published:** 2023-09-30

**Authors:** Shanta Das, Ajran Kabir, Chandra Shaker Chouhan, Md. Ahosanul Haque Shahid, Tasmia Habib, Md. Alamgir Kobir, Md. Zawad Hossain, Marzia Rahman, K. H. M. Nazmul Hussain Nazir

**Affiliations:** 1 Department of Microbiology and Hygiene, Faculty of Veterinary Science, Bangladesh Agricultural University, Mymensingh, Bangladesh; 2 Department of Medicine, Faculty of Veterinary Science, Bangladesh Agricultural University, Mymensingh, Bangladesh; 3 Department of Anatomy and Histology, Faculty of Veterinary Science, Bangladesh Agricultural University, Mymensingh, Bangladesh

**Keywords:** Pet cat, *S. aureus*, PCR, AMR, MDR

## Abstract

**Objective:**

This study sought to determine the occurrence, molecular identification, antimicrobial-resistant trends, and gene distribution of *Staphylococcus aureus* in pet cats and their owners’ hand swabs.

**Materials and Methods:**

From different places and clinics in Mymensingh and Dhaka, 168 pet cat samples and 42 hand swab samples from cat owners were obtained. The organisms were scrutinized by assessing the outcomes using conventional and molecular techniques. The disc diffusion technique was applied to find the resistance pattern against 12 antibiotics, and genes were discovered by targeting specific genes using PCR.

**Results:**

The occurrence of pathogenic *S. aureus* in pet cats was 7.74%, while it was 9.50% in pet owners’ hand swabs, and 25.0% of the pet owner’s hand swabs contained these genes. *Staphylococcus aureus* was utterly resistant to amoxicillin, ampicillin, cefixime, erythromycin, and imipenem in both pet cat and hand swabs of pet owner samples. All *S. aureus *isolates had a multidrug-resistant phenotype, and 1 from pet cats (O19) and 1 from pet owner hand swabs (H9) were resistant to all 12 antibiotics in the 7 antimicrobial classes. Several antibiotic-resistance genes were detected by PCR.

**Conclusion:**

The study confirmed multidrug-resistant pathogenic *S. aureus* in pet cats and their owners in Bangladesh, indicating a major health risk to both people and cats. Thus, a holistic and integrated one-health approach between veterinary and medical specialists is needed to mitigate the global distribution of these zoonotic antibiotic-resistant *S. aureus* strains.

## Introduction

*Staphylococcus aureus*, a commensal microorganism of animal and human microbial populations, is common in the respiratory system and skin [1]. *Staphylococcus aureus* may survive for a long time on hands and surfaces after initial exposure [2] and can act as an opportunistic pathogen in immune-compromised individuals [3]. It can trigger an assortment of infections, such as food poisoning, skin diseases, wound colonization, respiratory tract infections, and uniquely induced clotting [4,5].

The rise of antimicrobial resistance (AMR) among numerous pathogens poses an imminent danger to public health. The terrifying effects of AMR are a matter of concern for governments worldwide. AMR poses a threat to modern medicine’s existence. As a result, common diseases or traumas can kill humans. AMR is also crucial to zoonosis control and prevention [6]. Indiscriminate use of antibiotics in animals raises the danger of drug-resistant zoonotic diseases, which are increasing rapidly among animals and humans [6].

Currently, methicillin- and vancomycin-resistant strains of *S. aureus* (MRSA and VRSA, respectively) have emerged as prevalent in clinical and community settings. MRSA constitutes the most prominent antibiotic-resistant pathogen, prompting dangerous, and challenging infections to treat [7,8]. VRSA is a serious threat to be concerned about, as vancomycin is frequently considered a last-resort antimicrobial for addressing MRSA infections [9].

Companion animals become people’s closest friends because they share an emotional bond with their owners while representing their social standards and physical well-being. According to the American Pet Products Association, 68% of American homes had a pet in 2016, of which around 90 million were dogs and 94 million were cats [10]. A random survey of 2,980 United Kingdom households in 2007 indicated that 31% of households had cats [11]. In Bangladesh, petting animals was not so popular a few years ago. However, petting animals, particularly cats and dogs, is becoming well-accepted in the country’s larger cities, especially for children’s and owners’ emotional and social status [12].

This trend has exposed the potential risk that these companion animals can spread zoonotic infections such as *S. aureus* [13]. MRSA has become a serious threat to veterinary practices because pets can be a repository for human MRSA infections, and hospital MRSA cases have increased dramatically during the last 10 years [14]. Previous studies have revealed that circulating MRSA clones in pets such as cats and dogs are comparable to those found in people, specifically hospital-acquired clones [14]. Additionally, MRSA can be transferred between companion animals and their owners [15].

The close contact between cats and humans in domestic settings provides ample opportunities for transmitting and exchanging these antibiotic-resistant bacteria. While MRSA and VRSA have been extensively studied in human populations, their presence in animals, particularly domestic cats and their owners, has received comparatively less attention [7]. To our best knowledge, so far in Bangladesh, no detailed research has been done on identifying zoonotic strains of *S. aureus* and their resistance gene detection in cats and owners. In light of these considerations, the current work aimed to examine the occurrence and molecular identification of *S. aureus* in pet cats and owners and determine their AMR patterns and molecular detection of antibiotic resistance genes.

## Materials and Methods

### Ethical approval

The study was carried out in line with the Animal Welfare and Experimental Ethics Committee guidelines at Bangladesh Agricultural University. The samples were obtained after getting the appropriate consent from cat owners and explaining the study‘s objective [Approval No.: AWEEC/BAU/2019(51)].

### Sample collection and study period

Overall, 42 households were randomly selected from diverse locations and clinics in Dhaka and Mymensingh districts, where all the pet cats‘ oral swab samples (*n = *168) and their owners‘ hand swabs (*n = *42) were included in this study. The microbiological study was conducted at BAU‘s Department of Microbiology and Hygiene, Faculty of Veterinary Science, Mymensingh-2202, from January 2019 to June 2020.

### Culture, Gram staining, and biochemical confirmation of S. aureus

The organisms were initially enriched in the nutrient broth. Selected media, such as mannitol salt (Himedia, India) agar, were used to isolate *S. aureus*. Selected colonies were subjected to several biochemical tests, including the basic sugar fermentation test (dextrose, sucrose, maltose, lactose, mannitol), the coagulase test, and the catalase test, based on their observable cultural and Gram straining features.

### Extraction of genomic DNA and PCR

The genomic DNA of biochemically positive *S. aureus *isolates was extracted using a simple boiling approach [16]. One colony of each isolate was inoculated into 200 µl of distilled water and then boiled for 10 min. Subsequently, the specimens were placed on ice for a few minutes to induce cold shock and then centrifuged at 10,000 rpm for 10 min. The supernatant was retrieved and utilized as a DNA template in a PCR reaction. Table 1 lists the primers adapted to the current study. Previously isolated *S. aureus* from our lab was used as a positive control [2], and PCR water was used as a negative control in this study. PCR products were resolved on a 1.5% agarose gel, stained with ethidium bromide, and photographed and viewed under UV light using a gel documentation system.

### Determination of the antimicrobial profile

Twelve commonly used antibiotics (HiMedia, India) for cats were selected to determine the antibiotic susceptibility profile. All the positive *S*.* aureus* isolates were tested for antibiotic susceptibility using the agar disc diffusion procedure per the guidelines set forth by the Clinical Laboratory and Standards Institute (CLSI) [17]. The antimicrobial assay was done on Mueller-Hinton agar, purchased from a commercial manufacturer (HiMedia, India). The used antimicrobial agents were amoxicillin (AMX, 30 µg); erythromycin (E, 15 µg); ampicillin (AMP, 10 µg); imipenem (IMP, 10 µg); cefuroxime (CXM, 30 µg); cefotaxime (CTX, 30 µg); cefixime (CFM, 5 µg); amikacin (AK, 30 µg); norfloxacin (NOR, 10 µg); chloramphenicol (C, 30 µg); ciprofloxacin (CIP, 5 µg); and azithromycin (AZM, 15 µg). CLSI‘s zone diameter interpretative standards were used to categorize the outcomes of antimicrobial susceptibility testing as susceptible, intermediate, or resistant. [17]. Our isolates were phenotypically resistant to three or more chemical classes of antibiotics, considered multi-drug resistance (MDR) [2].

**Table 1.** List of primers with sequences used in this study.

Primer name	Gene targeted	Primer sequences (5'–3')	Amplicon size (bp)	Reference
*nuc* F	*nuc*	5'-GCG ATT GAT GGT GAT ACG GTC-3'	279	[18]
*nuc* R		5'-AGC CAA GCC TTG ACG AAC TAA AC-3'		
*bla_TEM_*-F	*bla_TEM_*	5'-CAT TTC CGT GTC GCC CTT AT-3'	793	[19]
*bla_TEM_*-R		5'-TCC ATA GTT GCC TGA CTC CC-3'		
*bla_ctx-M_*-F	*bla_CTX-M_*	5'-ATG TGC AGY ACC AGT AAR GTK ATG GC-3'	593	[19]
*bla_ctx-M_*-R		5'-TGG GTR AAR TAR GIS ACC AGA AYC AGC GG-3'		
*bla_SHV2_*-F	*bla_SHV2_*	5'-TTC GCC TGT GTA TTA TCT CCC TG-3'	854	[20]
*bla_SHV2_*-R		5'-TTA GCG TTG CCA GTG YTC G-3'		
*vanA*-F2	*VanA*	5'-AAT GTG CGA AAA ACC TTG CG-3'	677	[21]
*vanA*-R2		5'-CCG TTT CCT GTA TCC GTC C-3'		
*vanB*-F2	*VanB*	5'-GCT CCG CAG CCT GCA TGG A-3'	463	[22]
*vanB*-R2		5'-ACG ATG CCG CCA TCC TCC T-3'		
*vanC1*-F	*VanC*	5'-GAA AGA CAA CAG GAA GAC CGC-3'	796	[22]
*vanC1*-R		5'-TCG CAT CAC AAG CAC CAA TC-3'		
*mecA*-F	*mecA*	5'-AAA ATC GAT GGT AAA GGT TGG-3'	533	[23]
*mecA*-R		5'-AGT TCT GGC ACT ACC GGA TTT TGC-3'		
*mecC*-P1	*mecC*	5'-GAA AAA AAG GCT TAG AAC GCC TC-3'	138	[23]
*mecC*-P2		5'-GAA GAT CTT TTC CGT TTT CAG C-3'		

### Resistant gene determination

PCR was then applied to phenotypically positive antimicrobial samples to identify resistant genes. The primers utilized are enumerated in Table 1. Following PCR, the product was depicted as per the procedure described before.

## Results

### Occurrence of S. aureus in pet cats and hand swabs of pet owners

**Figure 1.**
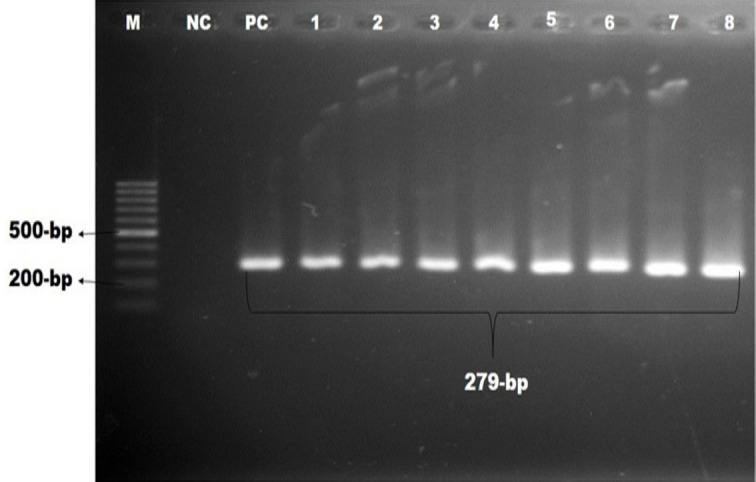
Identification of *S. aureus* by polymerase chain reaction. Gel electrophoresis showing *nuc *genes amplicons of *S. aureus* (279-bp). In Lanes: M-100 bp DNA ladder (Promega, USA), PC: positive control, NC: negative control, Lanes 1–8: complies with the samples of *S. aureus* showing approximately 279-bp.

On culture, Gram staining, and basic sugar fermentation biochemical assays, 32 of 168 pet cat isolates and 30 of 42 pet owner hand swabs showed positive for *Staphylococcus *spp. These isolates were confirmed as *S. aureus* by the coagulase test and further confirmed by amplifying the *nuc *gene (amplicon size 279 bp), as illustrated in Figure 1. The overall occurrence was 7.74% in pet cat samples and 9.52% in hand swabs of pet owners based on the coagulase test and PCR (Table 2).

### Molecular detection of antibiotic resistance genes from the isolates of pet cats and pet owners‘ hand swabs

**Figure 2.**
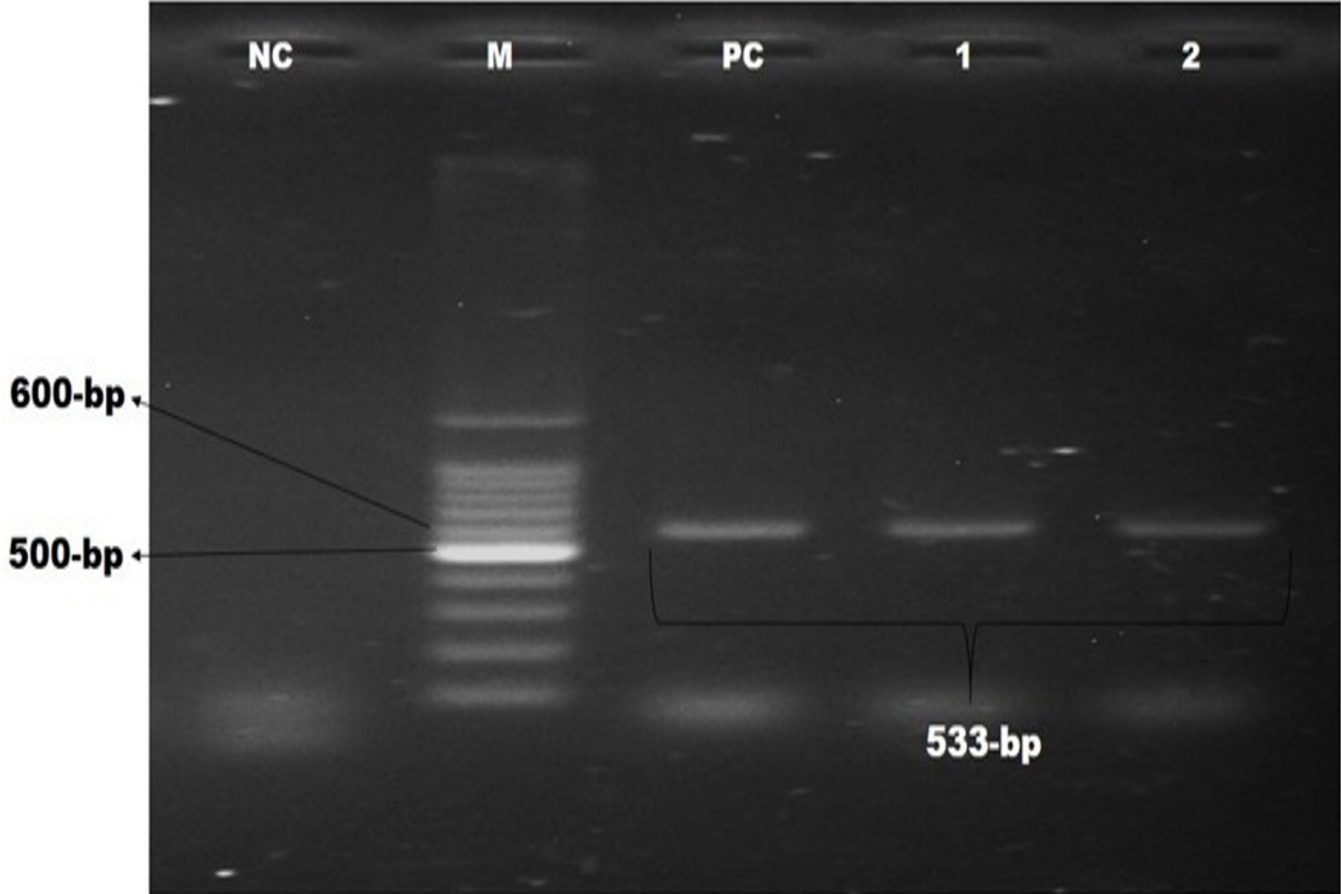
Identification of MRSA by polymerase chain reaction. Gel electrophoresis showing *mecA *genes amplicons of *S. aureus* (533-bp). In Lanes: M-100 bp DNA ladder (Promega, USA), PC: positive control, NC: negative control, Lanes 1–3: complies with the samples of MRSA showing approximately 533-bp.

**Figure 3.**
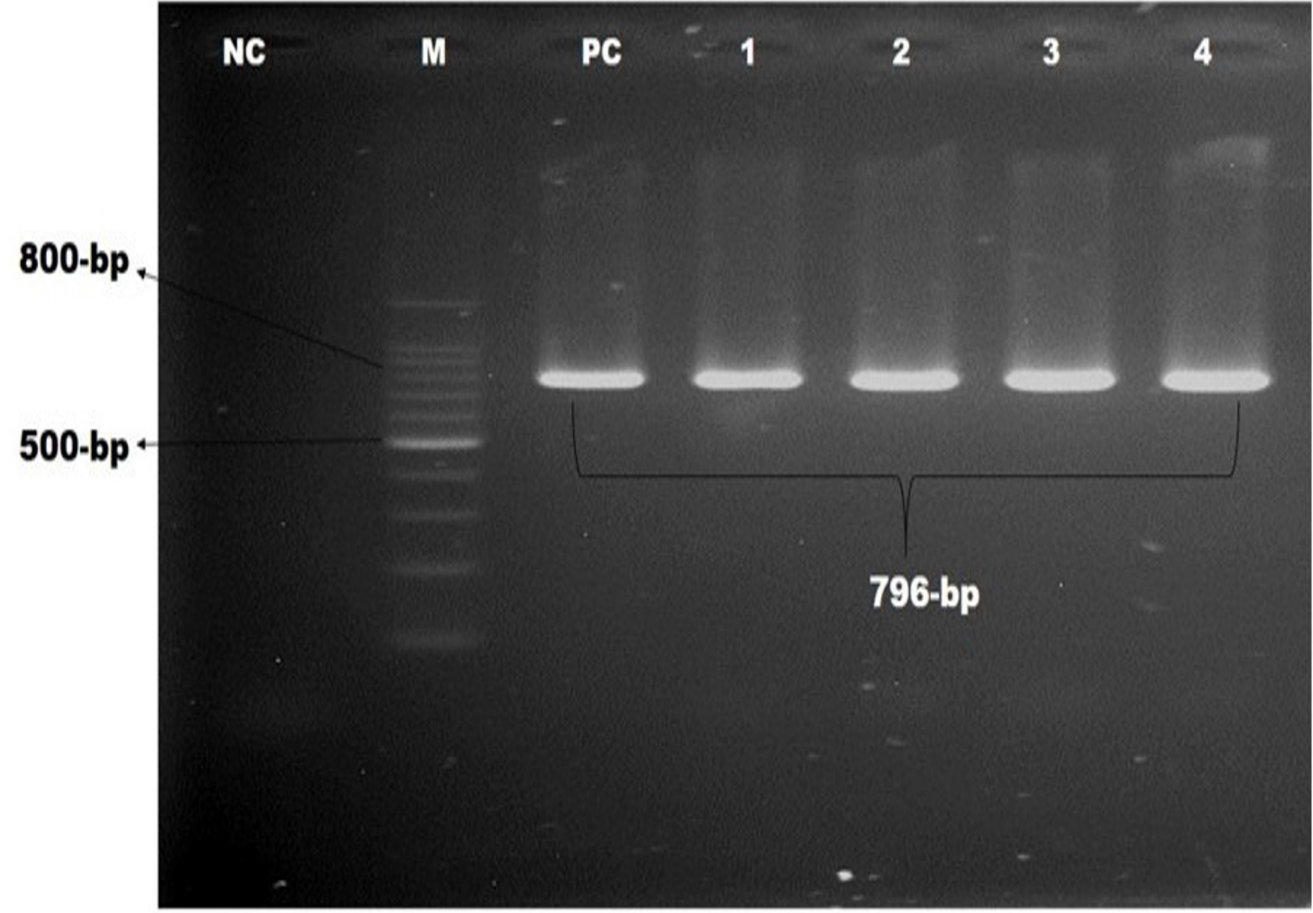
Identification of vancomycin-resistant gene of *S. aureus* by polymerase chain reaction. Gel electrophoresis showing *vanC *genes amplicons of *S. aureus* (796-bp). In Lanes: M-100 bp DNA ladder (Promega, USA), PC: positive control, NC: negative control, Lanes 1–4: complies with the samples of VRSA showing approximately 796-bp.

**Figure 4.**
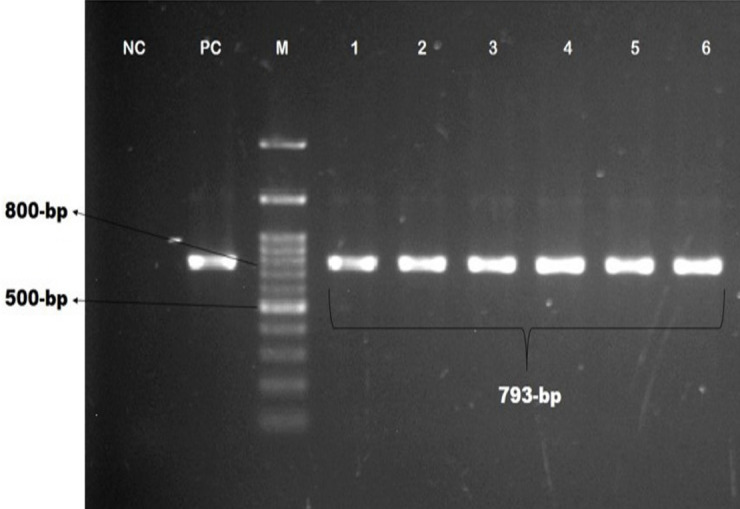
Identification of β-lactamases-producing *S. aureus* by polymerase chain reaction. Gel electrophoresis showing *bla_TEM_* genes amplicons of *S. aureus* (793-bp). In Lanes: M-100 bp DNA ladder (Promega, USA), PC: positive control, NC: negative control, Lanes 1–6: complies with the samples of β-lactamases-producing *S. aureus *showing approximately 793-bp.

**Figure 5.**
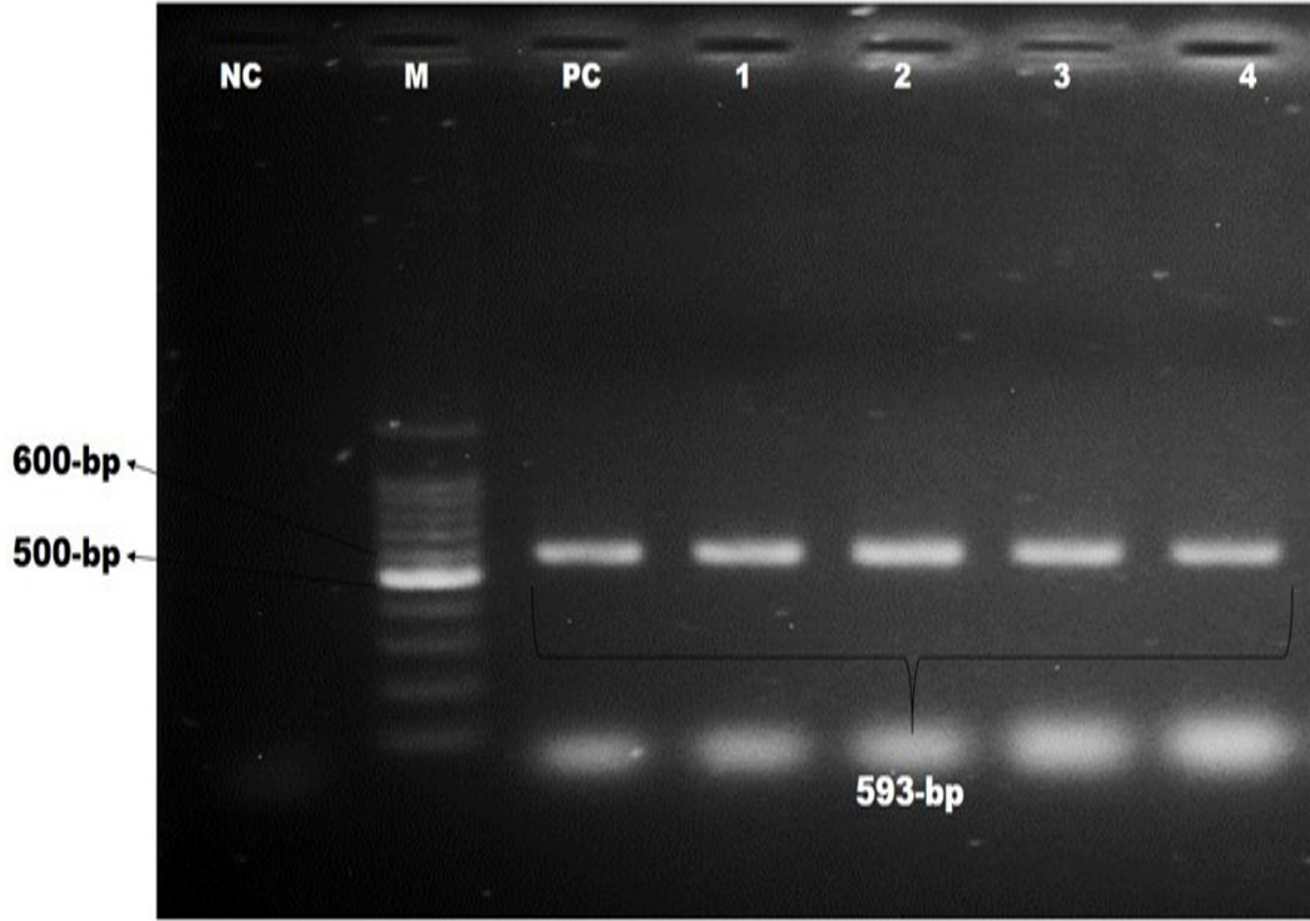
Identification of β-lactamases-producing *S. aureus* by polymerase chain reaction. Gel electrophoresis showing *bla_CTX-M _*genes amplicons of *S. aureus* (593-bp). In Lanes: M-100 bp DNA ladder (Promega, USA), PC: positive control, NC: negative control, Lanes 1–4: complies with the samples of β-lactamases-producing *S. aureus *showing approximately 593-bp.

**Figure 6.**
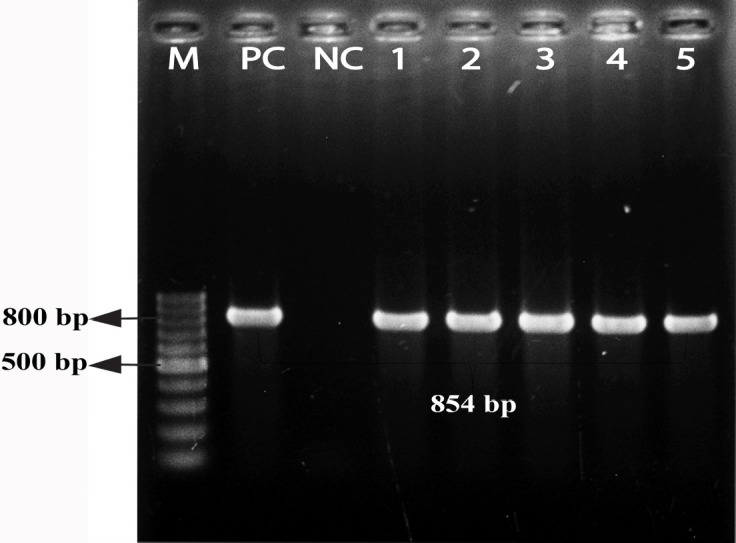
Identification of β-lactamases-producing *S. aureus* by polymerase chain reaction. Gel electrophoresis showing *bla_SHV-2 _*genes amplicons of *S. aureus* (854-bp). In Lanes: M-100 bp DNA ladder (Promega, USA), PC: positive control, NC: negative control, Lanes 1–5: complies with the samples of β-lactamases-producing *S. aureus *showing approximately 854-bp.

This study screened methicillin-resistance genes, including *mecA* and *mecC*, and vancomycin-resistant genes, including *vanA*,* vanB*, and *vanC. *It was observed that 15.4% of the pet cat isolates were positive for *mecA* genes, whereas 25% of this gene was in the hand swabs of their owner. Besides, *bla_TEM_*, *bla_CTX-M_*, and* bla_SHV2 _*genes were detected among these isolates described in Table 3. Molecular detection of *mecA*,* vanC*,* bla_TEM_*, *bla_CTX-M_*, and* bla_SHV2 _*genes is documented in Figures 2–6, respectively.

**Table 2.** Occurrence of *S. aureus *from pet cats and pet owners‘ hand swabs.

No.	Group	No of the total samples	No. of cultural, Gram staining biochemical *Staphylococcus* spp. positive samples	No. of Coagulase test and *nuc*- gene positive samples	Occurrence of *Staphylococcus* spp. (%)	Occurrence of *S. aureus* (%)
1.	Pet cat	168	32	13	40.62	7.74
2.	Hand swabs of pet owner	42	30	4	13.33	9.52

**Table 3.** Distribution of methicillin and vancomycin resistance genes of *S. aureus* from pet cats and pet owners‘ hand swabs.

Groups	No. of *Nuc*-positive samples	No. of resistant genes				
		*mecA*	*vanC*	*bla_TEM_*	*bla_CTX-M_*	*bla_SHV2_*
Pet cat	13	2 (15.4%)	2 (15.4%)	5 (38.46%)	2 (15.4%)	4 (30.76%)
Hand swabs of pet owner	4	1 (25.0%)	1 (25.0%)	1 (25.0%)	1 (25%)	2 (50%)

### Antibiogram profile of S. aureus from pet cats and pet owners‘ hand swabs

**Figure 7.**
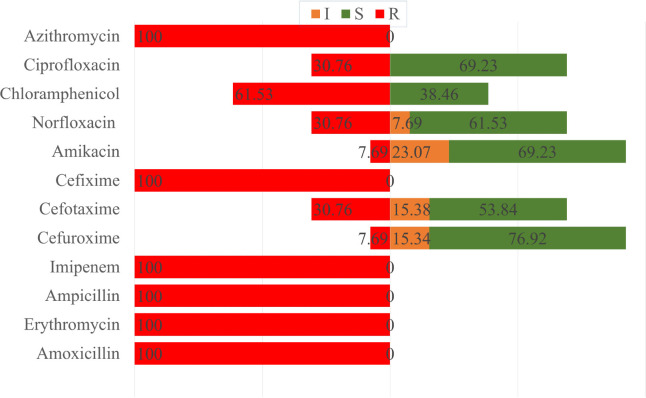
Antibiotic susceptibility patterns of pet cat *S. aureus* isolates.

**Figure 8.**
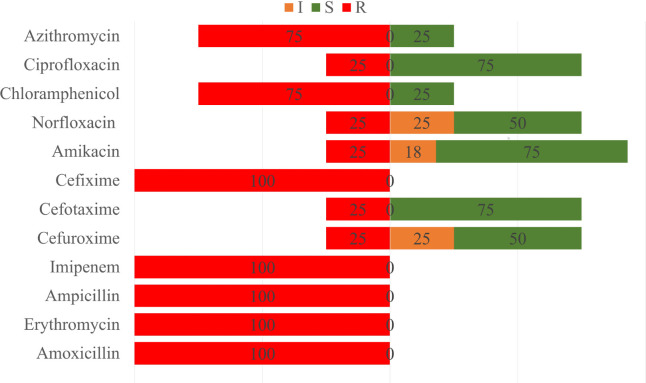
Antibiotic susceptibility patterns of owners hand swab *S. aureus* isolates.

Amoxicillin, ampicillin, cefixime, erythromycin, and imipenem resistance were 100% in both pet cat and hand swabs of pet owner-positive *S. aureus *samples. In samples from pet cats, azithromycin was resistant to 100% and chloramphenicol (61.5%) but sensitive to cefuroxime (76.92%) and amikacin (69.23%) (Figure 7). Subsequently, hand swabs from pet cat owner samples exhibited 75% resistance to chloramphenicol and azithromycin, whereas they were 75% sensitive to cefotaxime, ciprofloxacin, and amikacin and 50% sensitive to cefuroxime and norfloxacin (Figure 8).

### Phenotypic MDR nature of S. aureus from pet cats and pet owners‘ hand swabs

All positive *S. aureus* were isolated from pet cats, and hand swabs from the pet owner revealed the MDR phenotype. One *S. aureus* was isolated from pet cats (O19), and 1 from hand swabs from the pet owner (H9) showed resistance to all 12 antibiotics of the 7 antimicrobial classes tested in this study (Table 4).

The most prevalent MDR phenotype in *S. aureus* isolated from pet cats was amoxicillin, ampicillin, cefixime, erythromycin, azithromycin, and imipenem (AMX-AMP-CFM–E–AZM–IMP) (100%), followed by amoxicillin, ampicillin, cefixime, erythromycin, azithromycin, imipenem, and chloramphenicol (AMX-AMP-CFM–E–AZM–IMP–C) (61.5%) (Table 4).

In comparison, the most prominent phenotypic resistance pattern among *S. aureus* isolated from pet owners’ hand swabs was amoxicillin, ampicillin, cefixime, erythromycin, and imipenem (AMX-AMP-CFM–E–IMP) (100%), followed by amoxicillin, ampicillin, cefixime, erythromycin, azithromycin, imipenem, and chloramphenicol (AMX-AMP-CFM–E–AZM–IMP–C) (75.0%) (Table 4).

## Discussion

The probability of zoonotic pathogens spreading from people to pets has been stated in published research, as pets live adjacent to their respective owners and share comparable surroundings. Since ancient times, pets have significantly transmitted zoonotic pathogens to humans [24]. *Staphylococcus aureus, *an opportunistic pathogen that usually resides in the skin and mucosa of healthy humans and animals, can produce various infections such as food poisoning, skin diseases, wound colonization, and respiratory infections, and has a unique ability to induce clotting [5].

In addition, MRSA is a major concern for public health and veterinary issues associated with the zoonotic bacterium. MRSA usually causes severe infectious disorders such as food poisoning and pustular dermatitis in cats and dogs and severe pyogenic skin and soft tissue infections, food poisoning, pneumonia, otitis media, and endocarditis in humans [25].

MRSA spreads infections from humans to animals via skin infections and other means. Besides, Bangladeshi researchers have found MRSA in the country’s dog and cat populations [7,26]. Therefore, a one-health approach is required to combat these zoonotic infections effectively.

Pathogenic *S. aureus* infections were more common in pet cats (7.74%) but only in 40.62% of samples that were positive for culture, Gram stain, and biochemical in the present study, which is alarming. Similarly, Bierowiec et al. [3] reported that domestic cats (14.17%) showed a greater prevalence of *S. aureus *than feral cats (8.3%). As a result, pet cats could be considered a major reservoir for pathogenic staphylococci infections, which have a potential risk of being transmitted to humans through zoonotic transmission.

On the contrary, the total number of owners’ hand swab isolates in the present study was 9.52% and 13.33% in cultural, Gram-staining, and biochemical-positive samples, which was a little bit higher than the number of humans who carried the bacteria in their noses (7.7) [27]. Bierowiec et al. [3] revealed that pet owners are at high risk of being affected by *S. aureus *due to close contact, which justifies the present study’s findings. However, the high prevalence of *S. aureus* in owners’ hands might be due to exposure to the nose or nasal secretion of the cat or close contact with the cat.

Moreover, all of the isolates recovered from pet cats were found in the oral cavity, implying a higher rate of oral transmission. Abdel-Moein and Samir [27] found a high rate of oral carriage of *S. aureus* in companion animals. As a result, there is a risk of contaminating foods, surroundings, and households by orally shedding microorganisms. These contaminations may occur due to direct interaction with infected animals or indirect contact with contaminated household items. Therefore, this indicates a greater risk of staphylococcal infection from the contaminated materials for people with close contact or pet owners [28].

In the antibiotic sensitivity test, amoxicillin, ampicillin, imipenem, erythromycin, and cefixime were fully resistant to the isolated *S. aureus* isolates. This was most likely due to the widespread usage of these antimicrobials in pet animals as a treatment. Cefuroxime was almost sensitive to both samples in the investigation, which was a favorable indicator because cefuroxime is the most effective antibiotic against *S. aureus* in cat therapy [29,30]. All were found to have almost identical antibiogram profiles. We also found that all *S. aureus *isolates were MDR based on antibiogram profiles, which is critical.

This study also investigated the distribution of bacterial resistance genes in *S. aureus* isolates. There were 15.4% of the pet cat isolates positive for *mecA* and *vanC *genes, while 25% of the genes were in the hand swabs of their owner. However, no isolates tested positive for the *mecC*, *vanA*, or *vanB* genes. On the other hand, β-lactamases-producing genes were also screened, where *bla_TEM_* positive isolates were 38.46% and 25%, *bla_CTXM_* positive isolates were 15.4% and 25%, and *bla_SHV-2_* positive isolates were 30.76% and 50% in pet cats and owners’ hands, respectively. The presence of these genes confirmed β-lactamases-producing *S. aureus. *The presence of five genes in our isolates O11, O19, and H9 indicates that these isolates may produce extended-lactamases and be resistant to vancomycin and methicillin [2,19,31,32]. Further research is necessary to corroborate the presence of extended-spectrum beta-lactamases-producing* S. aureus, *VRSA, and MRSA*.*

Furthermore, all resistance genes were shown to be more prevalent in pet cats than in owners, which could be related to the availability and indiscriminate use of antibiotics in pet cats. Antibiotic-resistant *S. aureus* strains pose a global public health concern [33–36]. This is also alarming because nearly all antibiotics given to cats are prescribed in human medicine. And those resistance genes can be transmitted down to the following generations of bacteria via vertical gene transfer and shared among various bacterial populations. As a result, the findings of this study are crucial for pet cats and humans because they demonstrate the incidence of antibiotic-resistant *S. aureus*, a global zoonotic and public health concern.

**Table 4.** Distribution of *S. aureus* antibiotic profiles and resistance genes from pet cats and pet owners‘ hand swabs.

Isolate	Resistant phenotypes	Resistant genotype
Antibiotic profiles of isolated *S. aureus* isolates from pet cats		
O10	AMX, AMP, CFM, E, AZM, IMP, C, CIP	N/A
O11	AMX, AMP, CFM, E, AZM, IMP	*bla_TEM, _bla_CTX-M_, bla_SHV2_, mecA, vanC*
O12	AMX, AMP, CFM, E, AZM, IMP, C	N/A
O16	AMX, AMP, CFM, E, AZM, IMP, C	*bla_TEM_, bla_SHV2_*
O17	AMX, AMP, CFM, E, AZM, IMP, NOR,	*bla_TEM_, bla_SHV2_*
O19	AMX, AMP, CFM, E, AZM, IMP, C, CXM, CTX, NOR, CIP, AK	*bla_TEM_, bla_CTX-M, _bla_SHV2_, mecA, vanC*
O21	AMX, AMP, CFM, E, AZM, IMP, CTX, CIP	N/A
O22	AMX, AMP, CFM, E, AZM, IMP, AK, NOR	*bla_TEM_*
O23	AMX, AMP, CFM, E, AZM, IMP, C	N/A
O24	AMX, AMP, CFM, E, AZM, IMP, C	N/A
O28	AMX, AMP, CFM, E, AZM, IMP, C, CIP	N/A
O34	AMX, AMP, CFM, E, AZM, IMP, C, CTX, NOR	N/A
O37	AMX, AMP, CFM, E, AZM, IMP, CTX	N/A
Antibiotic profiles of isolated *S. aureus* isolates from pet cats owners hand swabs		
H8	AMX, AMP, CFM, E, IMP	N/A
H9	AMX, AMP, CFM, E, IMP, AZM, C, CXM, CTX, NOR, CIP, AK	*bla_TEM, _bla_CTX-M_, bla_SHV-2, _mecA, vanC*
H17	AMX, AMP, CFM, E, IMP, AZM, C	N/A
H22	AMX, AMP, CFM, E, IMP, AZM, C, CXM	N/A

O = oral, H = hand, AMX = amoxicillin, E = erythromycin, AMP = ampicillin, IMP = imipenem, CXM = cefuroxime, CTX = cefotaxime, CFM = cefixime, AK = amikacin, NOR = norfloxacin, C = chloramphenicol, CIP = ciprofloxacin, AZM = azithromycin.

## Conclusion

According to our findings, *S. aureus* was commonly found on pets and owners‘ hands. Antibiotic resistance was detected in many isolates, and these bacteria could potentially transmit resistance to their owners. Multidrug-resistant staphylococci were shown to be spread mostly through oral transmission in this investigation. Again, methicillin- and vancomycin-resistant genes were detected by PCR, which increases the chances of MRSA and VRSA among the pet population. Therefore, a comprehensive and integrated one-health approach, including public health and veterinary specialists, must combat the zoonotic transmission of antibiotic-resistant *S. aureus* between pets and their owners.

## List of abbreviations

BAU, Bangladesh Agricultural University; MDR, Multi-Drug Resistance; AMR, Antimicrobial Resistance; MRSA, methicillin-resistant *Staphylococcus aureus*; VRSA, Vancomycin-resistant *Staphylococcus aureus*.
